# Maternal and Fetal Tuberous Sclerosis: Do We Know Enough as an Obstetrician?

**Published:** 2017

**Authors:** Nalini Sharma, Shriram Sharma, Jion Lalnunnem Thiek, Santa Singh Ahanthem, Arnab Kalita, Donboklang Lynser

**Affiliations:** 1- Department of Obstetrics and Gynecology, North Eastern Indira Gandhi Regional Institute of Health and Medical Sciences Shillong, Meghalaya, India; 2- Department of Neurology, North Eastern Indira Gandhi Regional Institute of Health and Medical Sciences Shillong, Meghalaya, India; 3- Department of Radiology, North Eastern Indira Gandhi Regional Institute of Health and Medical Sciences Shillong, Meghalaya, India

**Keywords:** Pregnancy, Tuberous sclerosis complex, Tuberous sclerosis

## Abstract

**Background:**

Tuberous sclerosis, also known as tuberous sclerosis complex (TSC), is a rare genetic condition that mainly causes hamartomas to develop in different parts of the body. TSC, an autosomal dominant trait with variable penetrance, can adversely affect maternal and fetal outcome.

**Case Presentation:**

In this paper, a case of maternal and fetal tuberous sclerosis having fetal cardiac rhabdomyoma detected in utero at 26 weeks was reported who subsequently had fetal demise at 31 weeks.

**Conclusion:**

Tuberous sclerosis is a rare genetic condition that mainly causes development of hamartomas. In tuberous sclerosis, a cardiac rhabdomyoma is the only sign that can be detected prenatally. In maternal tuberous sclerosis, fetal ECHO is advisable after 24 weeks. A pregnancy complicated by maternal or fetal tuberous sclerosis deserves careful observation and the fetus should undergo prenatal fetal Doppler echocardiography and if possible magnetic resonance imaging for evaluation of other fetal structures including brain and renal parenchyma, so that parents can be counseled regarding its future prognostic implications. Tuberous sclerosis can lead to poor fetal outcome including intrauterine fetal death; hence regular antenatal follow up is required. Genetic counseling is recommended for couples who have a family history of tuberous sclerosis and who want to have children. Prenatal diagnosis is available for families with a known gene mutation or history of this condition.

## Introduction

Tuberous sclerosis, also known as tuberous sclerosis complex (TSC), is a rare genetic condition that causes mainly development of hamartomas in different parts of the body. TSC, an autosomal dominant trait with variable penetrance, can adversely affect maternal and fetal outcome.

This case is presented because of its rarity (tuberous sclerosis with pregnancy) and it can adversely affect maternal and fetal outcome. The obstetrician should know about this entity.

## Case Presentation

**Figure 1.**
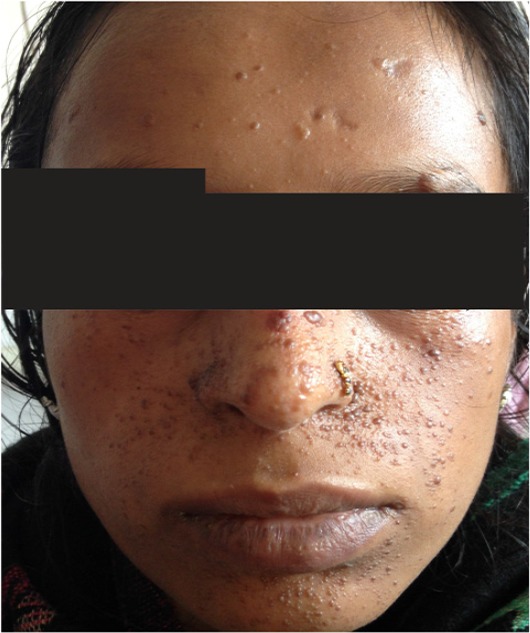
Picture showing adenoma sebaceum

**Figure 2.**
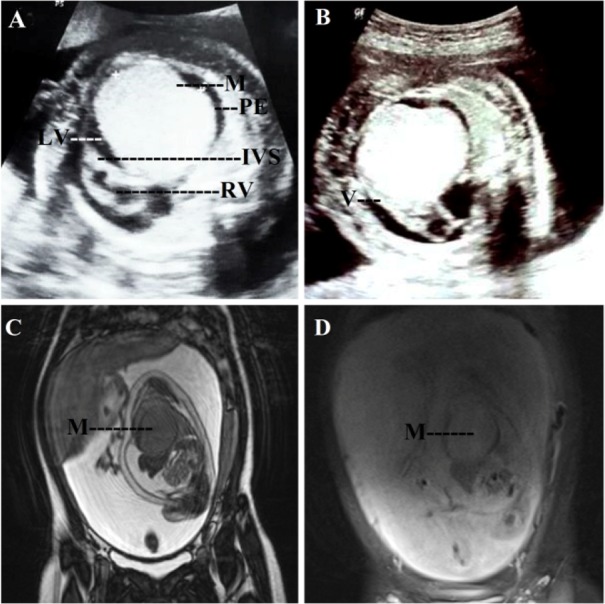
A 23 year old primi of tuberous sclerosis with live intrauterine fetus at 26 weeks of gestation showing cardiac rhabdomyoma. A: Ultrasound in ventricular diastole showing an echogenic mass (M) arising from the wall of the left ventricle (LV), note the pericardial effusion (PE), normal right ventricle (RV) and interventricular septum (IVS). B: Ultrasound showing the ventricle (V) in systole. C: MRI-T2WI showing an isointense mass (M) arising from the left ventricle. D: MRI with fat suppression, note that the mass (M) shows no visible fat suppression

**Figure 3.**
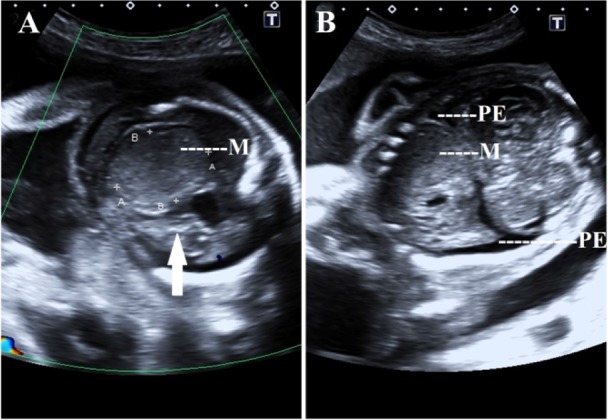
A 23 year old primi of tuberous sclerosis with fetal cardiac rhabdomyoma at 31 weeks of gestation resulting in IUFD. A: Color Doppler ultrasound showing absent cardiac activity (arrow), note the mass (M) and subcutaneous edema. B: Ultrasound in coronal section showing the mass (M) arising from the left ventricle, note the bilateral pleural effusion (PE)

A 23 year old primigravida was referred to the antenatal outpatient department (OPD) for the first time at 10 weeks of gestation (May, 2016). She was a known case of tuberous sclerosis complex. She was on antiepileptic medication. The last episode of seizure was 5 years ago. There was no family history of tuberous sclerosis. On examination, adenoma sebaceum (Figure 1) was present. General physical examination and systemic examination findings were normal except for the presence of systolic murmur on auscultation. The computed tomography scan (CT scan) of brain performed before pregnancy showed multiple parenchymal/perinuclear subependymal calcification, focal encephalomalacia. Echocardiography (ECHO) revealed a small patent ductus arteriosus (PDA) of 2 *mm* with left to right shunt. Fundoscopic examination revealed no abnormality. All the other routine antenatal investigations were normal. She was on regular follow up with regular consultation with neurologist. The anomaly scans which were performed at 13 and 20 weeks of gestation revealed no gross congenital anomaly. At 27 weeks of gestation while performing ultrasound for assessment of fetal growth, fetal cardiac rhabdomyoma (4×3.5 *cm*) (Figure 2) with other features of fetal hydrops (ascites, pericardial effusion, scalp edema, abdominal wall edema) was detected. Fetal cardiac rhabdomyoma was confirmed by fetal ECHO and MRI. Detailed antenatal anatomic survey using ultrasound ruled out angiolipoma of kidney and cerebral hamartoma. Cardiologist and cardiothoracic surgeon opinion was taken. Genetic counseling was done and patient was informed that the fetus was probably affected with tuberous sclerosis. The patient was recognized with intrauterine fetal demise by the antenatal OPD at 31 weeks of gestation (Figure 3). Induction of labour was done and the patient delivered a male, macerated baby weighing 1.2 *kg*. Intrapartum and peripartum period was uneventful. She was discharged after 48 *hr* of delivery with the advice to refer to postnatal clinic, and neurology OPD later.

## Discussion

Tuberous sclerosis, also known as tuberous sclerosis complex (TSC), is a rare genetic condition that mainly causes development of benign tumors in different parts of the body. The tumors most often affect the brain, skin, kidneys, heart, eyes and lungs. In the past, it was believed that the typical presentation included seizure, mental retardation, and facial angiofibroma (adenoma sebaceum) (Vogt’s Triad). This disorder has now wide variability of expression. Patients with TSC may be asymptomatic and of normal intelligence. TSC affects all races without a clear-cut predominance. TSC affects both sexes equally and it can present at any age. Its prevalence is around 1 in 6000–9000 individuals (1).

The inheritance of TSC is an autosomal dominant trait with variable penetrance. Tuberous sclerosis is caused by mutations in either the TSC1 or TSC2 gene. These genes are involved in regulating cell growth, and the mutations lead to uncontrolled growth and multiple tumors throughout the body. Only one of the genes needs to be affected for TSC to be present. The TSC1 gene is located on chromosome 9 and is called the hamartin gene. The other gene, TSC2, is located on chromosome 16 and is called the tuberin gene (1, 2).

It can be inherited from one parent with TSC or can result from a spontaneous genetic mutation. Children have a 50 percent chance of inheriting TSC if one of their parents has this condition. At this point, only one-third of TSC cases are known to be inherited. The other two-thirds result from a spontaneous and unpredictable mutation occurring during conception or very early development of the human embryo. In infant cardiac involvement and seizure are common presenting signs where dermatological, pulmonary or renal involvement may lead to diagnosis in older individuals. It can adversely impact fetal and maternal health. Pregnancy can be complicated by preeclampsia, intrauterine growth retardation, preterm labour, pre-term premature rupture of membrane, oligohydramnios, polyhydramnios, hydrops, abruption, haemorrhage from rupture renal tumor, renal failure and fetal demise (3, 4). Fetus may have rhabdomyoma and/or intracranial tubers (4). However, in one of the case series, fetal outcome was good (5) because none of the fetuses were affected by tuberous sclerosis.

In mother, complications of neurological involvement are the most common causes of mortality and morbidity. These are due chiefly to intractable epilepsy, status epilepticus, and subependymal giant cell astrocytoma (SEGA) with associated hydrocephalus. Renal complications are the next most frequent cause of morbidity and death. Less common are cardiac arrhythmias (which can present with sudden, unexplained death), congestive heart failure, and end-stage lung disease. Renal involvement appears to be the single most important prognostic factor in pregnancies with tuberous sclerosis. Renal evaluation should be performed in any patient who presents for preconception counseling. In tuberous sclerosis, a cardiac rhabdomyoma is the only sign that can be detected prenatally by ultrasound. In maternal tuberous sclerosis, fetal ECHO can be advisable after 22 weeks. A pregnancy complicated by maternal or fetal tuberous sclerosis deserves careful observation and the fetus should undergo prenatal fetal Doppler echocardiography and if possible an MRI for evaluation of other fetal structures including brain and renal parenchyma, so that parents can be counseled regarding its future prognostic implications (6).

Fetal cardiac rhabdomyomas are often benign and have a tendency to regress. It can occasionally induce poor outcome and the need for surgery depends on the patient’s clinical presentation (7). Diagnosis is usually made on an obstetric ultrasonography between 21 to 30 weeks. In our case, it was diagnosed at 26 weeks by obstetric ultrasonography and fetal ECHO. Tuberous sclerosis can lead to poor fetal outcome including intrauterine fetal death; hence regular antenatal follow up is required. Genetic counseling is recommended for couples who have a family history of tuberous sclerosis and who want to have children. Prenatal diagnosis is available for families with a known gene mutation or history of this condition. However, tuberous sclerosis often appears as a new DNA mutation. These cases are not preventable.

## Conclusion

Tuberous sclerosis is a rare genetic condition that mainly causes development of hamartomas. It can adversely affect maternal and fetal outcome. In tuberous sclerosis, a cardiac rhabdomyoma is the only sign that can be detected prenatally. In maternal tuberous sclerosis, fetal ECHO is advisable after 22 weeks.

A pregnancy complicated by maternal or fetal tuberous sclerosis deserves careful observation and the fetus should undergo prenatal fetal Doppler echocardiography and if possible an MRI for evaluation of other fetal structures including brain and renal parenchyma, so that parents can be counseled regarding its future prognostic implications. Tuberous sclerosis can lead to poor fetal outcome including intrauterine fetal death; hence regular antenatal follow up is required. Genetic counseling is recommended for couples who have a family history of tuberous sclerosis and who want to have children. Prenatal diagnosis is available for families with a known gene mutation or history of this condition.
